# Executive functions–based reading training engages the cingulo-opercular and dorsal attention networks

**DOI:** 10.1162/netn_a_00335

**Published:** 2023-12-22

**Authors:** Nikolay Taran, Rola Farah, Carmel Gashri, Ester Gitman, Keri Rosch, Bradley L. Schlaggar, Tzipi Horowitz-Kraus

**Affiliations:** Educational Neuroimaging Group, Faculty of Education in Science and Technology, Faculty of Biomedical Engineering, Technion Israel Institute of Technology, Haifa, Israel; Kennedy Krieger Institute, Baltimore, MD, USA; Department of Psychiatry and Behavioral Science, Johns Hopkins University School of Medicine, Baltimore, MD, USA; Department of Pediatrics, Johns Hopkins University School of Medicine, Baltimore, MD, USA; Department of Neurology, Johns Hopkins University School of Medicine, Baltimore, MD, USA

**Keywords:** Dyslexia, Executive functions, Visual attention, Intervention, fMRI, Functional connectivity

## Abstract

The aim of this study was to determine the effect of a computerized executive functions (EFs)–based reading intervention on neural circuits supporting EFs and visual attention. Seed-to-voxel functional connectivity analysis was conducted focusing on large-scale attention system brain networks, during an fMRI reading fluency task. Participants were 8- to 12-year-old English-speaking children with dyslexia (*n* = 43) and typical readers (*n* = 36) trained on an EFs-based reading training (*n* = 40) versus math training (*n* = 39). Training duration was 8 weeks. After the EFs-based reading intervention, children with dyslexia improved their scores in reading rate and visual attention (compared to math intervention). Neurobiologically, children with dyslexia displayed an increase in functional connectivity strength after the intervention between the cingulo-opercular network and occipital and precentral regions. Noteworthy, the functional connectivity indices between these brain regions showed a positive correlation with speed of processing and visual attention scores in both pretest and posttest. The results suggest that reading improvement following an EFs-based reading intervention involves neuroplastic connectivity changes in brain areas related to EFs and primary visual processing in children with dyslexia. Our results highlight the need for training underlying cognitive abilities supporting reading, such as EFs and visual attention, in order to enhance reading abilities in dyslexia.

## Author Summary

The aim of the study was to determine the behavioral and neural effects of a computerized executive functions (EFs)-based reading intervention. A total of 79 participants (8–12-year-olds, English-speaking) with and without dyslexia trained either on an EFs-based reading training or a math training. After the EFs-based reading intervention, children with dyslexia improved their scores in reading rate and visual attention. Intervention-related increases in fMRI functional connectivity were observed between the cingulo-opercular network and occipital regions. Higher indices of connectivity were related to better speed of processing and visual attention. The reading improvement involved neuroplastic connectivity changes in brain areas related to EFs and primary visual processing. The importance of training the cognitive abilities supporting reading in dyslexia (e.g., EFs and visual attention) is highlighted.

## INTRODUCTION

### Dyslexia: Definition and Explanatory Theories

Developmental dyslexia (henceforth, dyslexia) is classified as one type of specific learning disorder, with different studies reporting a prevalence between 5 and 20% (Norton et al., 2014; Schulte-Körne, 2010; Wagner et al., 2020). This disability is a heritable, life-long condition with early onset (Snowling, Hulme, & Nation, 2020). Dyslexia is described as a difficulty in accurate and fluent word recognition and spelling (Peterson & Pennington, 2012) that cannot be explained by sensorial deficits, insufficient literature exposure, delayed development of cognitive abilities, or low intelligence (Schulte-Körne, 2010).

For the last several decades, the scientific consensus regards dyslexia as a language disorder in which, for alphabetic-based written language, the proximate cause is a phonological processing deficit (Peterson & Pennington, 2012, p. 3). A close relationship exists between children’s phonological skills (i.e., phonological awareness) and the mastering of word reading (Melby-Lervåg, Lyster, & Hulme, 2012). According to the mentioned theory, children with reading difficulties manifest a neural processing deficit in the representation of the sounds in language. More recently, the role of executive functions (EFs) in dyslexia has been highlighted (Horowitz-Kraus, 2014; Varvara, Varuzza, Sorrentino, Vicari, & Menghini, 2014). EFs are a set of higher order cognitive abilities (inhibition, switching, updating; see (Miyake et al., 2000) that allow individuals to adapt and overcome different challenging environmental conditions (Diamond, 2020; Welsh, Pennington, & Groisser, 1991). Recent studies have highlighted the critical role EFs have in intact and impaired reading as supporting all three components of the Simple View of Reading model (SVR): word decoding (Kieffer & Christodoulou, 2020; Nguyen, Del Tufo, & Cutting, 2020), comprehension (Washburn, 2022), and critically, reading fluency—defined as fast and accurate reading (Silverman, Speece, Harring, & Ritchey, 2013). The three main EFs (inhibition, switching, updating; Miyake & Friedman, 2012) seem to have a direct effect on reading fluency (Kieffer & Christodoulou, 2020; Nguyen et al., 2020).

Children with dyslexia show dysfunctions in both verbal and visuospatial working memory, switching, and in the inhibition of irrelevant information (Barbosa, Rodrigues, Mello, Silva, & Bueno, 2019; Booth, Boyle, & Steve, 2010; Horowitz-Kraus, 2014; Varvara et al., 2014). Furthermore, children with dyslexia display below-average performance in speed of processing (Booth et al., 2010), which raised a theory regarding a slow speed of processing and a lack of synchronization between visual and auditory sensory modalities in these readers (Breznitz, 2006; Breznitz & Misra, 2003). Lastly, visuospatial attention difficulties were also reported (Franceschini, Gori, Ruffino, Pedrolli, & Facoetti, 2012; Varvara et al., 2014). These different theories aiming to explain the source of reading difficulties in dyslexia emphasize the complexity of the reading process, as outlined in the SVR (Hoover & Gough, 1990) and its refined extensions (Catts, 2018; Spencer, Richmond, & Cutting, 2020).

Dyslexia presents thus, a multifaceted nature with tight ontogenetic relation between the underlying neural systems (Dehaene, 2009), and variability related to the different types of orthographies (Siok, Jia, Liu, Perfetti, & Tan, 2020).

### Neurobiological Correlates of Dyslexia

The neural circuits associated with word decoding (the factor underlying the reading comprehension deficit in dyslexia, in terms of the SVR) encompass mostly left hemisphere areas, including the left inferior frontal gyrus or left IFG (Norton, Beach, & Gabrieli, 2015), inferior occipitotemporal regions (e.g., the Visual Word Form Area or VWFA), and regions around the tempo-parietal junction, including the angular (AG) and supramarginal gyri (SMG). Studies in adults report an engagement of the VWFA not only in decoding tasks (Cutting et al., 2013; Dehaene & Cohen, 2011), but also in phonological tasks (Conant, Liebenthal, Desai, Seidenberg, & Binder, 2020; Yarkoni, Speer, Balota, McAvoy, & Zacks, 2008), providing support for a role of this region in linking phonology and orthography. The SMG was found to be activated during auditory processing of syllabic sequences (Deschamps & Tremblay, 2014) as well as word reading (Weiss, Katzir, & Bitan, 2016). The processing features of the mentioned areas seem to be especially relevant for the decoding (i.e., visual-to-phonological translation) of graphemes and words (Vogel, Petersen, & Schlaggar, 2014). However, the specific mechanistic contributions of each of these cortical regions to the process of reading are not yet fully understood.

**Figure 1.**
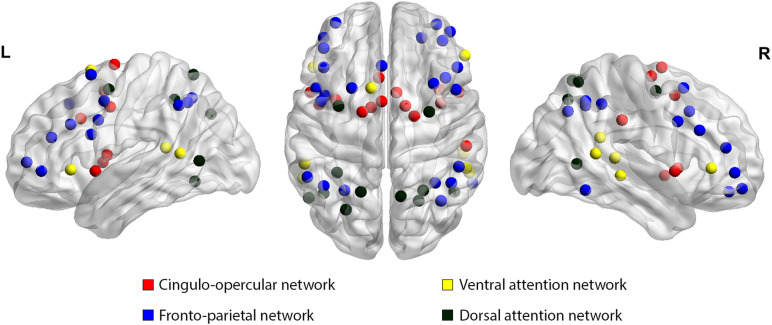
Graphical representation of the CO, the FP, the VAN, and the DAN, as described based on anatomical and functional meta-analyses (Power et al., 2011). From left to right: lateral view of the left hemisphere, superior view of the brain, lateral view of the right hemisphere.

It has been suggested that the functional connectivity of four different cognitive networks comprising the attention system are affected in dyslexia: the cingulo-opercular (CO), fronto-parietal (FP), ventral and dorsal attention networks (VAN and DAN, respectively) (Corbetta & Shulman, 2002; Farah, Ionta, & Horowitz-Kraus, 2021; Freedman, Zivan, Farah, & Horowitz-Kraus, 2020; Taran et al., 2022). The FP network seems to predispose to switching and goal-directed behavior, while the CO is related to error monitoring and feedback control (Dosenbach et al., 2007; Gratton, Sun, & Petersen, 2018). The VAN and DAN are mainly involved in visuospatial attention, in both bottom-up and top-down processes (see Figure 1) (Corbetta & Shulman, 2002; Liu, Bengson, Huang, Mangun, & Ding, 2016; Szczepanski & Kastner, 2013). We have previously suggested that artificially accelerated reading versus reading at a natural reading speed in English-speaking 8- to 12-years-old children with dyslexia is associated with increased synchronization between the CO-FP networks and visual-auditory networks (Horowitz-Kraus et al., 2022). These results echoed findings from Hebrew-speaking adults showing that during natural reading speed, there is a reduced synchronization between event-related potentials (ERPs) associated with visual and auditory processing, which was also associated with reduced speed of processing during word reading, supporting the asynchronization theory (Breznitz, 2006; Breznitz & Misra, 2003). These findings support the neural noise hypothesis in children with dyslexia as an explanation for their slow reading speed (Hancock et al., 2017), but also open up possibilities for reading improvements, especially in the fluency domain, to reduce this “noise.”

Crucially, the VWFA displays specific patterns of connectivity with the angular gyrus and the IFG, making it a candidate to be one of the links between linguistic (temporal language network) and attentional circuitry (FP) (L. Chen et al., 2019; López-Barroso et al., 2020; Stevens, Kravitz, Peng, Tessler, & Martin, 2017). This region is functionally connected to the DAN and is highly involved in general visual processing (Vogel, Miezin, Petersen, & Schlaggar, 2011). Furthermore, the angular gyrus and the IFG seem to play a role in language processing and reading as integrative, multimodal hubs, that is, recruiting and synchronizing large-scale whole-brain networks (Rosselli, Ardila, & Bernal, 2015; Taran et al., 2022; Xu, Lin, Han, He, & Bi, 2016). In summary, convergent evidence points toward the mentioned brain networks and regions as the primary neurobiological correlates of reading.

### Reading Interventions for Dyslexia

In line with the traditional, phonological understanding of dyslexia, most of the current interventions are focused on the explicit training of phonological awareness and grapheme-phoneme correspondence skills or spelling ability (Galuschka et al., 2020). Although these interventions generally show a positive effect on reading efficiency in children (Morris et al., 2012; Savage, Georgiou, Parrila, & Maiorino, 2018), investigating the effectiveness of phonological-based interventions suggests that additional subcomponents of reading can be trained in order to improve reading abilities (Snowling, 2013; Snowling et al., 2020; Strong, Torgerson, Torgerson, & Hulme, 2011; Williams, Walker, Vaughn, & Wanzek, 2017). Multiple empirical as well as review studies, meta-analyses, and theoretical works suggest that future treatments of dyslexia ought to be multisensorial (Snowling & Hulme, 2012), and focused not only on explicit phonological skills but also underlying cognitive abilities, such as EFs, speed of processing, and visuospatial attention skills (Horowitz-Kraus, Cicchino, et al., 2014; Peters, De Losa, Bavin, & Crewther, 2019; Stein, 2019; Vidyasagar, 2019). Furthermore, novel perspectives on reading instruction and remediation highlight the importance of improving sublexical skills and general aspects of language and knowledge in combination with phonological training (Fletcher, Savage, & Vaughn, 2021).

In the past years, several studies have demonstrated the effect of an EFs-based reading intervention targeting working memory, inhibition, visuospatial attention, and speed of processing while exposed to written materials (i.e., sentences) (Breznitz et al., 2013; Cecil, Brunst, & Horowitz-Kraus, 2021; Horowitz-Kraus, 2013; Horowitz-Kraus & Breznitz, 2014; Horowitz-Kraus & Holland, 2015; Horowitz-Kraus & Breznitz, 2014; Horowitz-Kraus, DiFrancesco, Kay, Wang, & Holland, 2015; Horowitz-Kraus, Toro-Serey, & DiFrancesco, 2015; Horowitz-Kraus, Vannest, et al., 2014) on reading, EFs, and brain structure and function. This training program forces the reader to visually follow the letters (engaging visual attention) as they are erased from the screen (reliance on working memory) and replaced by asterisks at a gradually increasing speed (speed of processing) without the ability to regress to the beginning of the sentence (inhibition) (Breznitz & Share, 1992; Cecil et al., 2021). This training was found to improve reading rate, accuracy and reading comprehension (Horowitz-Kraus, Cicchino, et al., 2014; Peters et al., 2019), as well as working memory, switching, shifting, visual attention, and speed of processing (Horowitz-Kraus & Breznitz, 2014; Horowitz-Kraus, Cicchino, Amiel, Holland, & Breznitz, 2014; Horowitz-Kraus, Hershey, Kay, & DiFrancesco, 2019; Peters et al., 2019). On the neurobiological level, this training was found to increase the connectivity strength between EF, attention, and sensory networks (visual processing and auditory networks) during word reading tasks (Horowitz-Kraus, DiFrancesco, et al., 2015; Horowitz-Kraus et al., 2019; Horowitz-Kraus & Holland, 2015); increase the within-network connectivity of the CO network during rest (Horowitz-Kraus, Toro-Serey, & DiFrancesco, 2015); increase the magnitude of error-detection ERPs during word reading errors (Horowitz-Kraus, 2016); and was associated with lower GLX/Glu concentration in the anterior cingulate cortex (Cecil, Brunst, & Horowitz-Kraus, 2021). It was suggested that the speeded deletion of letters from the screen (artificially inducing fluent reading), engages EFs and attention neural circuits and hence reduces the asynchrony/neural noise in the visual-auditory circuits (Cecil et al., 2021). However, the effect of this EFs-based reading intervention both behaviorally and neurobiologically compared to active control training, is yet to be resolved. More specifically, the effect of this EFs-based reading training on brain network connectivity during a reading fluency task against an active control group remains unknown.

### Aims and Hypotheses

The goal of the present study was to determine the neurobiological systems underlying the reading improvement following EFs-based reading intervention while performing a contextual reading fluency task. In the same vein, we aimed to measure the effects of the hypothesized neuroplastic changes on specific cognitive domains. To this end, functional MRI was acquired while children with dyslexia and typically reading children performed a contextual reading fluency task before and after the intervention. The experimental design included an active control group that trained using the math program.

Children with and without dyslexia were included in the experiment, given that the goal of the study was to explore the behavioral and neurobiological effect of the EFs-based reading training on both typical readers (TR) and children with dyslexia. Specifically, it was hypothesized that children with dyslexia would benefit more from the intervention both in terms of behavioral measures (visual attention, EFs, reading) and neurobiological changes, ultimately leading to a reduction in cognitive and functional connectivity disparities between the two groups.

We expected both TRs and children with dyslexia who underwent the EFs-based reading intervention to improve their contextual reading fluency, EFs, and attention abilities compared to those undergoing the control intervention. Neurobiologically, we hypothesized that higher functional connectivity in both TR and children with dyslexia would be found between EFs networks (FP, CO), attention networks (VAN, DAN), and sensory networks (visual, auditory) after the EFs-based reading intervention, relative to the improvement with math training. Additionally, children with dyslexia when compared with typical readers were expected to display greater changes both in functional connectivity (stronger connections) and behavioral performance (higher scores).

## METHODS

### Study Procedure

The studies were conducted at Cincinnati Children’s Hospital Medical Center, Ohio, USA. The experimental procedure was designed in accordance with the Declaration of Helsinki; it was reviewed and approved by the institutional review board. Participants’ parents signed informed consent before enrolling to the study. Children were compensated for their participation in the study (overall US$150). Exclusion criteria included comorbidity with attention difficulties, intellectual disability, or any other neurodevelopmental disorders, neurological or psychiatric conditions.

**Figure 2.**
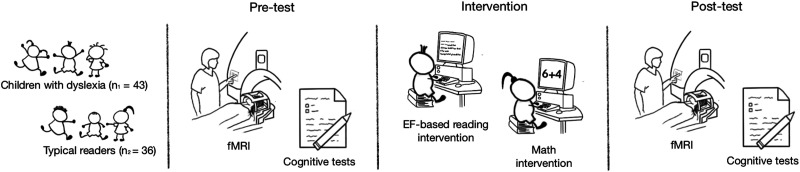
Seventy-nine children participated in the research project. All participants underwent behavioral assessment and resting-state fMRI scanning. Subsequently, they were randomly divided into two intervention groups: the executive functions (EFs)–based reading training (experimental group) and math training (active control group). After the intervention sessions, they underwent the same behavioral tests performed in the pretest and a second resting-state fMRI session.

First, all participants underwent a battery of cognitive tests. fMRI data were acquired while performing the contextual reading fluency task (see Figure 2 for an overview of the experimental design). Subsequently, all children were randomly divided into two intervention groups. One group underwent an EFs-based reading training, another group underwent a control (math) training. Both interventions had a duration of 8 weeks (3 sessions per week, 20–25 minutes per session). Finally, a posttest cognitive test battery was administered and a second fMRI session was conducted.

### Participants

A total of 79 English-speaking children participated in the experiment: 43 typical readers (TR, mean age = 10.04 ± 1.45, 18 females), and 36 children with dyslexia (children with dyslexia, mean age = 9.3 ± 1.36, 22 females). Participants from both groups were randomly divided into two intervention groups: one group underwent the EFs-based reading training (21 TR, 19 children with dyslexia) and the second group performed computerized math training (22 TR, 17 children with dyslexia), which served as an active control measure. There were no significant differences in nonverbal reasoning abilities between the reading groups (TR mean percentile = 67.27 ± 19.33, mean percentile = 56.83 ± 22.82, *t*(77) = 2.20, *p* > .05) nor the intervention groups (EFs-based reading training group mean percentile = 59.9 ± 21.9, math training group mean = 62.67 ± 19.9, *t*(77) = .581, *p* > .05). Similarly, there were no significant differences between the groups in age or sex (children with dyslexia mean age = 9.65 ± 1.42, TR mean age = 9.96 ± 1.34, *t*(77) = 1.23, *p* > .05; EFs-based reading training group mean age = 9.8 ± 1.4, math training group mean age = 9.6 ± 1.4, *t*(77) = .329, *df* = 77, *p* > .05). The present study experienced a sample attrition rate of 16.8%: 16 out of 95 participants did not complete the training (6 dropped out from the EFs-based reading intervention and 10 from the math intervention) and were subsequently excluded from further analysis. The final number of participants (79) provided a statistical power above 95% for both independent samples *t* tests and 2 × 2 repeated measures ANOVA (Rosner, 2011).

### Behavioral Measures

#### General cognitive abilities.

Nonverbal intelligence was evaluated using the Test of Nonverbal Intelligence (TONI) (Brown, Sherbenou, & Johnsen, 2010). General verbal abilities (receptive vocabulary) were evaluated using the Peabody Picture Vocabulary Test, 4th edition (PPVT-4) (Dunn & Dunn, 2007).

#### Reading abilities.

Reading abilities in both TR and children with dyslexia were evaluated using a battery of normative English tests: orthographic processing: Test of Word Reading Efficiency (TOWRE-Sight Word Efficiency) (Torgesen, Wagner, & Rashotte, 1999); phonological processing: Comprehensive Test of Phonological Processing (CTOPP) (Wagner, Torgesen, Rashotte, & Pearson, 2013); reading accuracy (number of correctly read words), reading rate (reading speed), and comprehension: Gray Oral Reading Test (GORT) (Wiederholt & Bryant, 2012); and Test of Silent Reading Efficiency and Comprehension (TOSREC) for reading comprehension (Schrank, Mather, & McGrew, 2014). Orthographic and phonological processing were measured using isolated word/nonword reading. Reading accuracy/rate were measured in contextual reading.

#### Executive functions and attention abilities.

Executive functions were assessed using several tasks designed to address the three main EFs: (1) working memory was assessed using both forward and backward digit recall as implemented in the Digits span subtest of the Wechsler Intelligence Scale for Children (WISC-IV) (Wechsler, 2011); (2) switching: using the letter-number sequencing subtest from the Trail Making Test from the Delis–Kaplan Executive Function System (DKEFS) (Delis et al., 2011); and (3) inhibition: using the Color-Word subtest (condition 3) of the DKEFS. Speed of processing was tested using the Coding and Symbol search subtests of the WISC.

Selective visual attention was assessed using the Sky-search subtest of the Test of Everyday Attention for Children (TEA-Ch) (Manly et al., 2001).

### Neuroimaging Data

#### Neuroimaging data acquisition.

The fMRI images were acquired using a Philips Ingenia 3 Tesla MRI scanner (Philips Healthcare, Best, Netherlands). Each fMRI session (pretest and posttest) was 13 minutes long and the repetition time (TR) was 1 second: a whole-brain T2* functional volume was acquired every 1 second for a total of 780 volumes per session. The echo time (TE) was 30 ms. A field of view (FOV) of 20 × 20 × 14.4 cm, matrix of 80 × 80, and slice thickness of 3 mm were utilized. In addition, for each participant, whole-brain T1 images were acquired in order to coregister the functional images to a high-resolution anatomical image. The TR for the T1 scan was 8.1 ms, with a TE of 3.7 ms, inversion time of 940 ms and a flip angle of 8°. The FOV was 22.4 × 25.6 × 16 cm, matrix of 224 × 256, and slice thickness of 1 mm.

Before the first fMRI session, all children were invited to explore the MRI scanner environment and to practice laying down on the scanner bed “as still as a statue” (Taran et al., 2022; Vannest et al., 2014). Foam pads were placed on either side of the head-coil apparatus in order to minimize motion. Presentation of the stimuli was possible using an MRI-compatible audiovisual system (Avotec, SS3150/SS7100).

#### Neuroimaging task.

**Figure 3.**
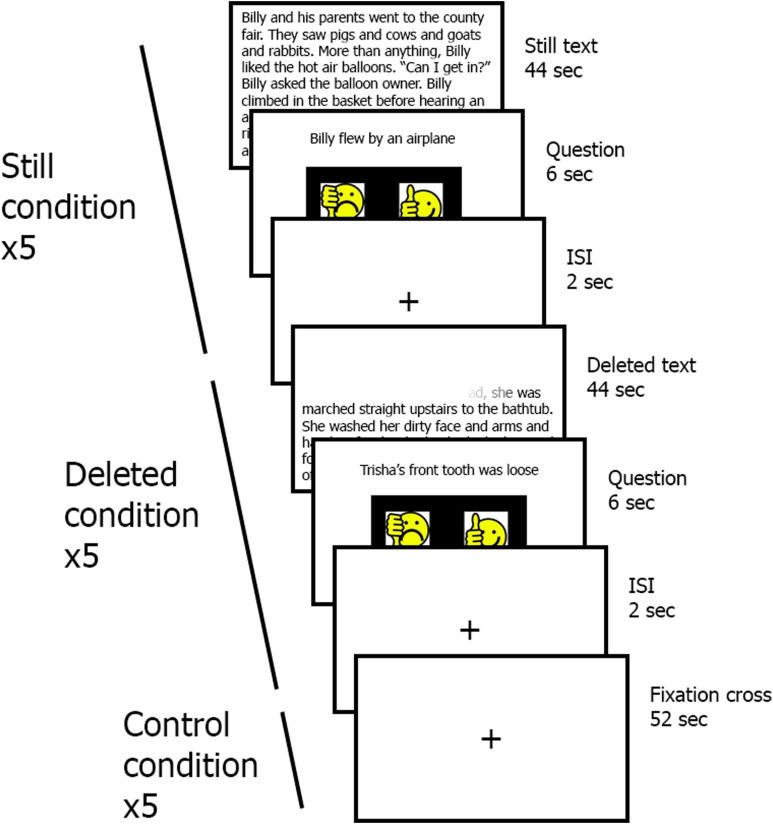
Graphical representation of the experimental fluency fMRI task (Taran et al., 2022). Three different conditions were presented in an interleaved fashion: Still, in which the participants were asked to read a Still text; Deleted, which consisted in accelerated reading; and a Control condition, consisting in a fixation cross presented in the middle of the screen. Each condition was presented 5 times in each session.

Participants performed the fluency task inside the scanner before and after the intervention (see Figure 3). The fluency task is a reading task including two different reading conditions: “Still condition,” in which a written story appears on the screen for 44 seconds, and “Deleted condition,” in which the presented text is deleted letter by letter starting from the first letter and is completely deleted after 44 seconds. After the story, participants are presented with a yes/no question based on the text. Response times and accuracy are recorded. The Deleted condition was developed based on accelerated reading in resemblance to the EFs-based reading intervention. A constant deletion rate of 119 ms per letter was utilized in the Deleted condition, which was previously reported to be the average reading rate of children with and without dyslexia between the ages of 8 and 12 (Horowitz-Kraus, Cicchino, et al., 2014; Taran et al., 2022). This reading rate was selected to ensure that all participants could read the text passages without encountering significant difficulties.

The written stories were between 200 and 250 characters in length. The fMRI experiment was divided into 15 blocks (5 Still stories, 5 Deleted stories, and 5 control blocks). The length of each block was 52 seconds: 44 seconds for the story, 6 seconds for reading and answering the question and a 2 second-long inter stimulus interval during which a fixation cross was presented in the center of the screen. The control condition consisted in a fixation cross presented in the center of the screen for 52 seconds. There were two different sets of stories, one for the preintervention fMRI session and another one for the postintervention fMRI session. The text passages presented in the preintervention and postintervention did not differ in difficulty: text difficulty was calculated based on sentence length, repetition of words, word length, and frequency (mean difficulty pretest ± *SD* = 602 ± 90, posttest = 654 ± 102; *t*(9) = 1.32, *p* = .22).

#### EFs-based reading training.

The EFs-based reading training was developed based on the finding that reading a text that is being deleted from the screen improves reading fluency in both TR and individuals with dyslexia—fluency defined as fast and accurate reading (Breznitz et al., 2013). Previous research suggests that this kind of training improves reading fluency by boosting different cognitive functions within the EFs domain such as working memory and inhibition, but also speed of processing and visual attention (Breznitz et al., 2013).

Here, children trained three times per week (for 8 weeks) for a total of 24 sessions: each session was 20 to 25 minutes long. In an initial session, the reading speed of each participant was calculated. This was done by presenting sentences on the screen (without deleting them), and once the participant finished reading the sentences, they were instructed to push the space bar, which prompted a multiple-choice comprehension question. Automated calculation of reading speed was performed by dividing the reading time of each text passage by the number of characters in that specific fragment of text. It is important to note that only the passages for which participants answered the reading comprehension questions correctly were included in this calculation. Once the reading pace was established, the training phase started with a series of text passages that were being erased from the screen at that individual’s reading speed. The training phase consisted in reading short sentences (~100 characters) and answering a multiple-choice reading comprehension question presented immediately after each sentence. The characters within the sentence were being deleted from the screen and replaced by asterisks starting from the first letter at a gradually increasing pace; the erasure rate would increase only when the participant’s answer to the reading comprehension question was correct on 10 consecutive trials (Cecil et al., 2021).

#### Math training (control group).

Math training was administered using an online math tool customized for each participant’s grade level. The training encompassed various topics for third- to sixth-grade participants, including number properties, operations, and more. Third-grade training covered number sense, addition, subtraction, multiplication, division, properties, mixed operations, fractions, geometry, data, and measurement. Fourth-grade training incorporated all of the previous topics, along with decimals and operations on fractions. Fifth-grade training included all previous topics, as well as exponents, powers of 10, data, measurement, and operations on decimals. Sixth-grade training covered all previous topics and more: number theory, estimations, ratios and rates, equations, percents, money, time, graphs, and statistics. The training platform was accessed through the website https://www.ixl.com/math. The intervention duration was similar to that of the EFs-based reading training: three sessions per week for 8 weeks (totaling 24 sessions), with each session lasting approximately 20 to 25 minutes. This training did not have a speed component in it.

### Data Analyses

#### Behavioral data.

In order to find baseline performance differences between TR and children with dyslexia, independent two-sided Student’s *t* tests were utilized. To determine intervention-related differences in the behavioral tests (outlined in the Behavioral Measures session), 2 Training (Reading, Math) × 2 Time (Pretest, Posttest) repeated measures ANOVA were conducted (Toffalini et al., 2021). These analyses were conducted for the whole group as well as for children with dyslexia and TR separately. All statistical analyses included age, sex, and socioeconomic status as covariates.

Raw *p* values were adjusted using Bonferroni’s procedure for multiple comparisons. Significance value (alpha) was set to 0.05. The statistical analysis was conducted using IBM SPSS statistics version 28 (IBM, 2021).

#### Neuroimaging data.

Image preprocessing was performed using Statistical Parametric Mapping 12 (SPM) (Friston, Penny, Ashburner, Kiebel, & Nichols, 2006) and CONN (version 20b) (Whitfield-Gabrieli & Nieto-Castanon, 2012). The preprocessing of the fMRI data included five different steps performed in order to adjust the fMRI volumes and to increase the signal-to-noise ratio. These steps included: (1) realignment and motion correction, (2) outlier identification, (3) segmentation, (4) normalization and coregistration, and (5) smoothing. Motion is an especially relevant noise source in young children (Power et al., 2011). Thus, additional motion correction tools were utilized: aComprCor method for nuisance regression was combined with scrubbing of consecutive functional volumes with global signal changes of intensity above z = 3 and/or framewise displacement above 0.5 mm, in line with recent benchmarking recommendations (Ciric et al., 2017). It was confirmed that there were no significant differences between the framewise displacements of the two groups. High-pass and low-pass filters were utilized in order to keep the fMRI signal of interest for the fluency task: high-pass filter 0.0096 Hz, low-pass filter 0.165 Hz (Bijsterbosch, Smith, & Beckmann, 2017). Only the Still and Deleted reading conditions were included in the analysis (specifically, only the 44 seconds during which text was being read, leaving out the time of question reading and response). The rest condition was not included in the analysis.

Seed-to-voxel analyses were conducted on the fMRI data. Boxcar hemodynamic response functions (HRFs) were generated to align with the distinct blocks comprising the experimental design. These functions were incorporated in the denoising and first-level analysis stages as implemented in CONN version 20b (Whitfield-Gabrieli & Nieto-Castanon, 2012) for the calculation of task-residual functional connectivity between the chosen seed regions and the rest of voxels in the brain (Tran et al., 2018). A previously described brain atlas developed based on anatomical and functional meta-analyses was utilized for the definition of regions and networks of interest (Power et al., 2011). The Power264 atlas includes 264 regions grouped in 14 networks. Four cognitive brain networks involved in visual attention processing and EFs were chosen as the seeds (i.e., the DAN, VAN, FP, and CO). One network was taken at a time and all of its nodes were defined as one seed. Subsequently, the correlation between the averaged time series of all voxels within the seed and every other voxel in the brain was computed, before exploring whether any brain region showed significant correlations with the seed (for single level models) or whether any significant change in connectivity existed between one condition and another (for multilevel models). The voxels were reconstructed in 2 × 2 × 2 mm for the analysis. Voxel-wise statistics throughout the whole brain were performed at an FDR-corrected cluster-level *p* < 0.05. This procedure yielded multiple statistical parametrical functional connectivity maps for each seed. All neuroimaging results were corrected for multiple comparisons.

The validity of the relations between neurobiological variables and behavioral traits drawn by brain-wide association studies (BWAS) has been questioned in a recent benchmarking paper (Marek et al., 2022). Even though the present paper cannot be considered a BWAS, it does report of univariate brain-behavior associations, the reliability of which seems to be lower than was initially thought (Marek et al., 2022). Here, a control permutation analysis was performed to increase the validity of the reported results: the sample (79 pre-post fMRI datasets) was randomly divided into four subgroups (resembling the four experimental groups in the original 2 × 2 [group] × condition [design]) before seed-to-voxel analysis using the DAN as a seed. The 79 participants were divided into four groups using the rand MATLAB command. Marek et al. (2022), conclude that a random division of the subjects into different subgroups could induce spurious results that can contradict the actual obtained results. Here, null results were expected when comparing the randomized groups.

#### Correlation between neuroimaging and behavioral data.

In order to evaluate the strength of the linear relationship between cognitive test results and functional connectivity values, correlative analyses were conducted. Functional connectivity values between different brain regions in which increases in connectivity strength were detected were tested as predictors of cognitive performance. This was calculated thus: first, for the group in which the increase was detected and second, for the whole sample. Linear correlation was examined using Pearson’s *r* correlation coefficient. Spurious significance resulting from multiple comparisons was controlled using Bonferroni’s correction.

Structural equation modeling-based moderation analyses were conducted as well. Specifically, the areas showing enhanced synchronization after the reading training in the neuroimaging analysis were tested as moderators of the improvement in reading performance.

## RESULTS

### Baseline Behavioral Measures (Pretest)

#### Reading and verbal abilities.

The TR group consistently scored higher across reading measures, including phonological awareness, word reading, pseudoword reading, and reading comprehension (see Table 1). No significant differences were found between groups in receptive vocabulary or nonverbal intelligence (PPVT-4 number of errors, *t* = .905, *p* = .36; TONI percentile, *t* = 1.8, *p* = .1).

**Table 1.** Cognitive abilities before intervention

**Ability**	**Test**	**TR group (*n* = 43)**		**Dyslexia group (*n* = 36)**		**Student’s *t* (*p* value)**	**Degrees of freedom**	**Cohen’s *d***
		**Mean**	***SD***	**Mean**	***SD***			
Phonological awareness	CTOPP Elision (Std. score)	11.35	1.94	8.00	2.77	6.11***	61.07	1.4
Phonological awareness	CTOPP Letter naming (Scaled score)	8.83	2.241	6.78	2.11	4.15***	76	0.94
Word reading	TOWRE - SWE (Scaled score)	104.37	11.14	81.94	12.52	8.42***	77	1.9
Pseudoword reading	TOWRE - PDE (Scaled score)	105.33	9.91	84.75	10.88	8.79***	77	1.9
Reading comprehension	TOSREC (Index)	104.07	17.46	85.64	14.49	5.04***	77	1.1
Reading comprehension	GORT Comprehension (Percentile)	65.68	26.89	27.27	22.27	7.10***	77	2
Nonverbal intelligence	TONI (Percentile)	66	19	55	21	1.8	77	0.2
Updating/Working memory	WISC Digit Span (Std. score)	11.4	2.72	9.72	2.17	2.98**	77	0.6
Switching	DKEFS Trail Making Test Condition 4 (Std. Score)	10.72	2.75	7.39	4.27	3.89***	51.56	0.9
Inhibition	DKEFS Color-Word Condition 3 (Std. Score)	10.65	2.76	8.18	3.42	3.53***	77	0.8
Visual attention	TEA-Ch Sky Search Attention (Percentile)	35.16	25.9	23.79	19.72	2.13*	73	0.5
Speed of processing	WISC Symbol Search (Std. score)	11.16	3.02	9.5	2.18	2.83**	75.48	0.6

*Note*. *SD* = standard deviation, TR = typical readers. Significant *p* values are reported following APA guidelines.

* *p* < 0.05.

** *p* < 0.01.

*** *p* < 0.001.

#### Executive function and attention abilities.

TR outperformed children with dyslexia in several EFs, including working memory, switching, and inhibition (see Table 1). Furthermore, there were significant differences between the groups in speed of processing and visual attention, with children with dyslexia displaying poorer performance.

### The Effect of the Intervention on Behavioral Measures (Pretest vs. Posttest)

#### Reading abilities.

Whole group analysis: ANOVA analysis of the pretest and posttest scores showed a significant main effect of Time for contextual reading rate (*F*(1, 78) = 5.64, *p* < .05, ηp^2^ = .07, see Table 2), suggesting overall improvement in reading rate between the pretest and posttest for all participants. No significant main effect of training was observed. Furthermore, the analysis revealed a significant Time × Training interaction for all participants (*F*(1, 78) = 4.88, *p* < .05, ηp^2^ = .06). This result suggests that children undergoing the EFs-based intervention improved their contextual reading rate more than those undergoing the math intervention, regardless of the presence of dyslexia. A partial eta squared of .06 and a Cohen’s *f* equal to .38 indicated a moderate-to-strong effect size. However, there was no significant Time, Training, nor Time × Training interaction effects on reading accuracy nor comprehension (when considering both children with dyslexia and TR together).

**Table 2.** The effect of the intervention on reading and cognitive abilities

Ability test	Group	Math int. pretest (mean, *SD*)	Math int. posttest (mean, *SD*)	EFs-based int. pretest (mean, *SD*)	EFs-based int. posttest (mean, *SD*)	Main effect of time	Main effect of training	Time * Training Interaction
Word reading **TOWRE SWE**	Whole sample (79)	93.9 (15.9)	93 (20)	94.4 (16.8)	95 (16.2)	*F*(1, 77) = .19, *p* = .66, η_p_^2^ = .002	*F*(1, 77) = .01, *p* = .9, η_p_^2^ = .001	*F*(1, 77) = .01, *p* = .91, η_p_^2^ = .001
	TR (43)	104.8 (9.2)	100.8 (22.8)	103.9 (13.1)	103.3 (14)	*F*(1, 41) = .66, *p* = .42, η_p_^2^ = .02	*F*(1, 41) = .05, *p* = .82, η_p_^2^ = .001	*F*(1, 41) = .36, *p* = .55, η_p_^2^ = .009
	DD (36)	79.9 (10.8)	73 (8.7)	83.7 (13.8)	85.7 (13.5)	*F*(1, 34) = 4.23, *p* = .04*, η_p_^2^ = .11	*F*(1, 34) = .73, *p* = .4, η_p_^2^ = .02	*F*(1, 34) = .18, *p* = .67, η_p_^2^ = .005
Pseudoword reading **TOWRE PDE**	Whole sample	96.6 (14.6)	94.9 (16.1)	95.3 (14.7)	94.3 (22.5)	*F*(1, 77) = .76, *p* = .38, η_p_^2^ = .01	*F*(1, 77) = .04, *p* = .83, η_p_^2^ = .001	*F*(1, 77) = .05, *p* = .83, η_p_^2^ = .001
	TR	105.5 (8.9)	105.7 (10.3)	105.1 (11.1)	105.7 (11)	*F*(1, 41) = .13, *p* = .73, η_p_^2^ = .003	*F*(1, 41) = .15, *p* = .69, η_p_^2^ = .004	*F*(1, 41) = .18, *p* = .67, η_p_^2^ = .004
	DD	85 (12.1)	81 (9.7)	84.5 (10)	84.5 (15.5)	*F*(1, 34) = 1.87, *p* = .18, η_p_^2^ = .05	*F*(1, 34) = 1.97, *p* = .17, η_p_^2^ = .005	*F*(1, 34) = .17, *p* = .68, η_p_^2^ = .005
Contextual reading rate **GORT Reading Rate**	Whole sample	41.9 (28.9)	41.7 (27.6)	41.1 (31.8)	49 (28.6)	*F*(1, 77) = 5.64, *p* = .04*, η_p_^2^ = .07	*F*(1, 77) = .395, *p* = .53, η_p_^2^ = .004	*F*(1, 77) = 4.88, *p* = .02*, η_p_^2^ = .06
	TR	57.3 (27.6)	61.6 (27.6)	58.8 (25.7)	65.8 (19)	*F*(1, 41) = .33, *p* = .57, η_p_^2^ = .008	*F*(1, 41) = .02, *p* = .93, η_p_^2^ = .001	*F*(1, 41) = 5.78, *p* = .02*, η_p_^2^ = .12
	DD	21.3 (20.3)	24.5 (18.2)	20.1 (14.8)	27.2 (20)	*F*(1, 34) = 5.9, *p* = .02*, η_p_^2^ = .15	*F*(1, 34) = .02, *p* = .93, η_p_^2^ = .001	*F*(1, 34) = .83, *p* = .37, η_p_^2^ = .03
Contextual reading accuracy **GORT Reading Accuracy**	Whole sample	50.9 (31.7)	50.1 (32.5)	48.8 (32.9)	49.4 (28.7)	*F*(1, 77) = .08, *p* = .78, η_p_^2^ = .001	*F*(1, 77) = .02, *p* = .88, η_p_^2^ = .001	*F*(1, 77) = .001, *p* = .97, η_p_^2^ = .001
	TR	70.2 (23.8)	70.9 (24)	73.3 (24.5)	65.6 (26.2)	*F*(1, 41) = 1.7, *p* = .19, η_p_^2^ = .04	*F*(1, 41) = .03, *p* = .88, η_p_^2^ = .001	*F*(1, 41) = 2.5, *p* = .17, η_p_^2^ = .06
	DD	26 (22)	23.7 (20.4)	25.1 (18.9)	32.3 (20.4)	*F*(1, 34) = 1.8, *p* = .18, η_p_^2^ = .05	*F*(1, 34) = .34, *p* = .56, η_p_^2^ = .01	*F*(1, 34) = 6.5, *p* = .01*, η_p_^2^ = .16
Reading comprehension **GORT reading comprehension**	Whole sample	50.3 (31.8)	47.5 (29.1)	46.5 (31.7)	45.2 (29.2)	*F*(1, 77) = 2.2, *p* = .14, η_p_^2^ = .03	*F*(1, 77) = .1, *p* = .75, η_p_^2^ = .001	*F*(1, 77) = .01, *p* = .91, η_p_^2^ = .001
	TR	65.8 (28.6)	61.8 (24.2)	68.2 (25.2)	62 (22.1)	*F*(1, 41) = 2.5, *p* = .12, η_p_^2^ = .06	*F*(1, 41) = .03, *p* = .86, η_p_^2^ = .001	*F*(1, 41) = .1, *p* = .75, η_p_^2^ = .003
	DD	30.1 (23.5)	29.1 (24.4)	27.4 (22.8)	27.4 (25.1)	*F*(1, 34) = .06, *p* = .8, η_p_^2^ = .002	*F*(1, 34) = .07, *p* = .79, η_p_^2^ = .002	*F*(1, 34) = .06, *p* = .8, η_p_^2^ = .002
Working memory **WISC Digit Span**	Whole sample	11.1 (2.8)	11.3 (2.9)	10.2 (2.3)	10.5 (3.3)	*F*(1, 76) = 1.1, *p* = .29, η_p_^2^ = .01	*F*(1, 76) = 1.99, *p* = .16, η_p_^2^ = .03	*F*(1, 76) = .01, *p* = .94, η_p_^2^ = .001
	TR	11.9 (3)	12.3 (3)	10.8 (2.3)	12.1 (3.2)	*F*(1, 40) = 5.72, *p* = .02*, η_p_^2^ = .13	*F*(1, 40) = .66, *p* = .19, η_p_^2^ = .02	*F*(1, 40) = 1.77, *p* = .19, η_p_^2^ = .04
	DD	9.5 (2.2)	8.7 (2.2)	9.9 (2.5)	10.1 (2.5)	*F*(1, 34) = .99, *p* = .33, η_p_^2^ = .03	*F*(1, 34) = 1.77, *p* = .19, η_p_^2^ = .05	*F*(1, 34) = 1.75, *p* = .19, η_p_^2^ = .05
Inhibition **DKEFS Color-Word**	Whole sample	9.7 (2.6)	10.5 (2.5)	9.5 (3.8)	10.5 (3.6)	*F*(1, 75) = 18.83, *p* < .001***, η_p_^2^ = .2	*F*(1, 75) = .05, *p* = .82, η_p_^2^ = .001	*F*(1, 75) = .08, *p* = .78, η_p_^2^ = .001
	TR	10.9 (2)	11.6 (1.9)	10.4 (3.4)	11.4 (2.9)	*F*(1, 41) = 9.3, *p* = .004**, η_p_^2^ = .18	*F*(1, 41) = .21, *p* = .65, η_p_^2^ = .005	*F*(1, 41) = .42, *p* = .52, η_p_^2^ = .01
	DD	7.9 (2.4)	9.2 (2.6)	8.5 (4.1)	9.5 (3.9)	*F*(1, 32) = 9.54, *p* = .004**, η_p_^2^ = .23	*F*(1, 32) = .15, *p* = .69, η_p_^2^ = .005	*F*(1, 32) = .083, *p* = .77, η_p_^2^ = .003
Switching **Trail Making Test**	Whole sample	9.5 (3.8)	10.8 (3.8)	9.1 (3.9)	10.2 (3.8)	*F*(1, 73) = 12.6, *p* < .001***, η_p_^2^ = .15	*F*(1, 73) = .95, *p* = .33, η_p_^2^ = .01	*F*(1, 73) = 1.16, *p* = .28, η_p_^2^ = .02
	TR	10.6 (3.2)	12 (2.8)	10.8 (2.2)	11.14 (3.3)	*F*(1, 41) = 3.8, *p* = .06, η_p_^2^ = .08	*F*(1, 41) = .19, *p* = .66, η_p_^2^ = .005	*F*(1, 41) = 1.4, *p* = .24, η_p_^2^ = .03
	DD	7.8 (4.2)	10.2 (3.5)	7.3 (4.4)	9 (3.9)	*F*(1, 30) = 8.89, *p* = .006**, η_p_^2^ = .23	*F*(1, 30) = .43, *p* = .52, η_p_^2^ = .01	*F*(1, 30) = .31, *p* = .58, η_p_^2^ = .01
Speed of processing **WISC Symbol Search**	Whole sample	10.3 (2.5)	11.5 (2.7)	10.5 (3.1)	11.4 (3.4)	*F*(1, 76) = 11.87, *p* < .001***, η_p_^2^ = .14	*F*(1, 76) = .007, *p* = .93, η_p_^2^ = .001	*F*(1, 76) = .12, *p* = .73, η_p_^2^ = .002
	TR	10.9 (2.6)	12.6 (2.8)	11.4 (3.5)	12.4 (3.7)	*F*(1, 41) = 10.64, *p* = .002**, η_p_^2^ = .21	*F*(1, 41) = .03, *p* = .86, η_p_^2^ = .001	*F*(1, 41) = .45, *p* = .51, η_p_^2^ = .01
	DD	9.6 (2)	10.1 (2)	9.5 (2.4)	10.3 (2.4)	*F*(1, 33) = 2.19, *p* = .15, η_p_^2^ = .06	*F*(1, 33) = .003, *p* = .96, η_p_^2^ = .001	*F*(1, 33) = .08, *p* = .78, η_p_^2^ = .002
Visual attention **TEA-Ch Sky search**	Whole sample	7.6 (3.1)	10 (6.4)	7.4 (2.7)	10 (3)	*F*(1, 72) = 29.4, *p* < .001***, η_p_^2^ = .29	*F*(1, 72) = .12, *p* = .73, η_p_^2^ = .002	*F*(1, 72) = .08, *p* = .78, η_p_^2^ = .001
	TR	8.2 (3.1)	11.3 (4.1)	8 (2.5)	10.6 (2.7)	*F*(1, 36) = 14.68, *p* < .001***, η_p_^2^ = .29	*F*(1, 36) = .57, *p* = .46, η_p_^2^ = .02	*F*(1, 36) = .77, *p* = .39, η_p_^2^ = .02
	DD	6.9 (3)	8.4 (3)	6.7 (2.7)	9.5 (3.4)	*F*(1, 34) = 17.53, *p* < .001***, η_p_^2^ = .34	*F*(1, 34) = .217, *p* = .64, η_p_^2^ = .006	*F*(1, 34) = 5.13, *p* = .02*, η_p_^2^ = .14

*Note*. Cohen’s *f* measure of effect size is reported for the significant Time × Training interactions. TR = typical readers; DD = developmental dyslexia; *F* = ANOVA *F* term.

* *p* < .05.

** *p* < .01.

*** *p* < .001.

Children with dyslexia: The results of the statistical analysis revealed significant effects of Time on word reading and reading rate for children with dyslexia, indicating that all participants with dyslexia improved their single word reading abilities and contextual reading speed, regardless of the administered intervention (*F*(1, 34) = 4.23, *p* < .05, ηp^2^ = .11, *F*(1, 33) = 5.9, *p* < .05, ηp^2^ = .15 for word reading and reading rate, respectively). Partial eta squared indices between .11 and .15 and Cohen’s *f* between .4 and .6 indicated a moderate-to-strong effect size. Furthermore, an analysis of variance conducted on reading accuracy in children with dyslexia revealed a significant interaction effect (Time × Training), indicating that participants with dyslexia who underwent EFs-based reading training improved their contextual reading accuracy scores more than those undergoing math training (*F*(1, 33) = 6.5, *p* < .05, ηp^2^ = .16). A partial eta squared of .16 and a Cohen’s *f* equal to .69 indicated a strong effect size.

Typical readers: Children without dyslexia who underwent the EFs-based reading training improved their performance in reading rate more than those undergoing math training, as revealed by a significant ANOVA interaction effect (Time × Training *F*(1, 41) = 5.78, *p* < .05, ηp^2^ = .12). A partial eta squared equal to .12 and a Cohen’s *f* equal to .38 indicated a moderate effect size.

#### EFs and attention abilities.

Whole group analysis: Significant main effects of Time were revealed for inhibition, switching, processing speed, and visual attention, indicating that all participants improved their performance, regardless of the training condition (*F*(1, 75) = 18.83, *p* < .001, ηp^2^ = .2; *F*(1, 76) = 11.87, *p* < .001, ηp^2^ = .14; and *F*(1, 76) = 11.87, *p* < .001, ηp^2^ = .14; and *F*(1, 72) = 29.4, *p* < .001, ηp^2^ = .29, respectively). However, there were no significant main effects of training nor was there evidence of differential effects of training condition for the reading difficulties and TR groups (see Table 2).

Statistical analyses of the pre-post scores on EFs tests revealed no significant differential effects of the training for the EFs-based reading training versus Math training on any EFs: working memory, switching, and inhibition were tested (see Table 2).

In order to test the effect of training on speed of processing, a Time × Training repeated measures ANOVA was conducted on WISC Symbol Search scores.

The effect of the EFs-based reading intervention on visual attention scores was not significant as tested by 2 × 2 repeated measures ANOVA: Sky search Time × Training interaction *F*(1, 72) = 3.01, *p* = .09).

Children with dyslexia: The subgroup of children with dyslexia displayed significant improvements in inhibition and switching over time regardless of the intervention group that the belonged to (as revealed by a significant ANOVA main effect of Time, see Table 2).

ANOVA analysis of visual attention scores revealed that the interaction term (Time × Training) was significant for children with dyslexia (*F*(1, 34) = 5.127, *p* < .05, ηp^2^ = .14), suggesting larger improvements in visual attention scores after the EFs-based reading intervention when compared to the active control math intervention. The observed effect size for children with dyslexia was large (partial eta squared equaled .14, Cohen’s *d* was equal to .46).

Typical readers: The subgroup of children without a diagnosis of dyslexia displayed significant increases in working memory performance, as revealed by a significant ANOVA main effect of Time (*F*(1, 40) = 5.72, *p* < .05, ηp^2^ = .13). Noteworthy, all participants displayed this improvement, regardless of the received treatment. Improvements in inhibition and speed of processing were apparent as well, as revealed by significant main effects of Time (*F*(1, 41) = 9.3, *p* < .01, ηp^2^ = .18 and *F*(1, 41) = 10.64, *p* < .01, ηp^2^ = .21, respectively).

### Fluency fMRI Task

#### Baseline (pretest).

Accuracy: TR outperformed children with dyslexia in both Still and Deleted conditions (see Table 3). The number of correct responses was statistically lower in children with dyslexia when compared to TR in both conditions. There were no significant differences between the children who later underwent EFs-based reading training and those in the math group (*p* > .05).

**Table 3.** Baseline performance in the fluency task; significant differences between children with dyslexia and TR

		Children with dyslexia	Typical readers	Student’s *t* (*p* value) Cohen’s *d*
Still	Correct	3.710 ± 1.29	4.27 ± 1.11	2.06, .02*
				Cohen’s *d* = .47
	Response time	4,136 ± 1,020	3,580 ± 961	2.47, .008*
				Cohen’s *d* = .56
Deleted	Correct	4.05 ± .97	4.79 ± .67	3.93, <.001***
				Cohen’s *d* = .89
	Response time	4,107 ± 856	3,422 ± 859	3.5, <.001***
				Cohen’s *d* = .79

*Note*. Independent samples *t* tests indicate significant differences between TR and children with dyslexia in the task. Cohen’s *d* measure of effect size is reported.

* *p* < .05.

** *p* < .01.

*** *p* < .001.

Reaction time: TR showed shorter response times when compared to children with dyslexia in both conditions (see Table 3). No significant differences were found between the intervention groups (EFs-based reading intervention vs. math intervention) in the baseline reaction time (*p* > .05).

#### Preintervention versus postintervention.

Accuracy: No significant differences in accuracy were found in any of the four subgroups. Two by two (2 × 2) repeated measures ANOVA—Time (pretest/posttest) × Training (EFs-based reading int./Math int.)—did not show any significant main effects nor significant interaction effects (see Table 4).

**Table 4.** Pre-post differences in fluency task performance

		Pretest				Posttest				Contrast	*T* test (*p* value), Cohen’s *d*
		Still		Deleted		Still		Deleted			
		Response time (ms)	Correct answers	Response time (ms)	Correct answers	Response time (ms)	Correct answers	Response time (ms)	Correct answers		
Typical readers	EFs-based reading intervention	3,553 ± 1,097 (A)	4.09 ± 1.37	3,330 ± 1,014	4.7 ± .9	4,147 ± 1,012 (B)	3.95 ± 1.09	3,236 ± 845	4.7 ± .47	B > A	3.15** Cohen’s *d* = .71
	Math intervention	3,606 ± 836 (C)	4.45 ± .8	3,510 ± 693	4.86 ± .35	4,055 ± 693 (D)	3.95 ± 1.14	3,296 ± 981	4.6 ± .94	D > C	2.5* Cohen’s *d* = .55
Children with dyslexia	EFs-based reading intervention	4,267 ± 1,019 (E)	3.47 ± 1.42	4,204 ± 721	4.1 ± .9	4,833 ± 965 (F)	3 ± 1	3,936 ± 953	4.6 ± .91	F > E	2.47* Cohen’s *d* = .63
	Math intervention	3,980 ± 1,032	4 ± 1.1	3,703 ± 971	4 ± .96	4,423 ± 1,239	3.33 ± 1.15	3,703 ± 971	4.58 ± .9		

*Note*. Paired *t* tests indicate higher response times in the fluency task after the EFs-based reading intervention. Cohen’s *d* measure of effect size is reported.

* *p* < .05.

** *p* <. 01.

*** *p* < .001.

Reaction time: Overall, all participants undergoing EFs-based reading intervention showed significantly greater response times to the Still condition reading comprehension questions after the intervention (see Table 4). Furthermore, TR (but not children with dyslexia) undergoing Math intervention showed increased response times to the Still condition comprehension questions in the posttest when compared to the pretest.

Longer response times in the posttest were associated with increases in accuracy, measured as the number of correct responses (Pearson’s *r* = −.43, *R*^2^ = .18, *p* < .001). In other terms, children demonstrating increases in response time to the Still condition (between the pretest and the posttest) displayed higher accuracies in the same condition (in the posttest). This association suggested a possible relation between the change in response times and the intervention-related change in EFs. In line with the a posteriori formulated hypothesis, a significant correlation was found between pre-post change in response time during the Still condition and pre-post change in inhibition in all participants (Pearson’s *r* = −.31, *R*^2^ = .09, *p* = .01). Specifically, the participants showing larger improvements in inhibition after the intervention displayed longer response times after the intervention.

### Neuroimaging Results

#### Baseline connectivity analysis (pretest).

Baseline differences found between children with dyslexia and TR in the connectivity patterns of attention-related brain networks were reported previously (Taran et al., 2022) and are outside the scope of the present paper, which focuses on intervention-related brain connectivity changes. For this reason, only pre-post results are presented here.

### The Effect of Intervention on Functional Connectivity (Pretest vs. Posttest): Visual Attention Networks

#### Dorsal attention network (DAN).

Still condition: No differences in brain connectivity while reading Still text were found in the DAN when comparing baseline and postintervention seed-to-voxel statistical parametric maps.

**Figure 4.**
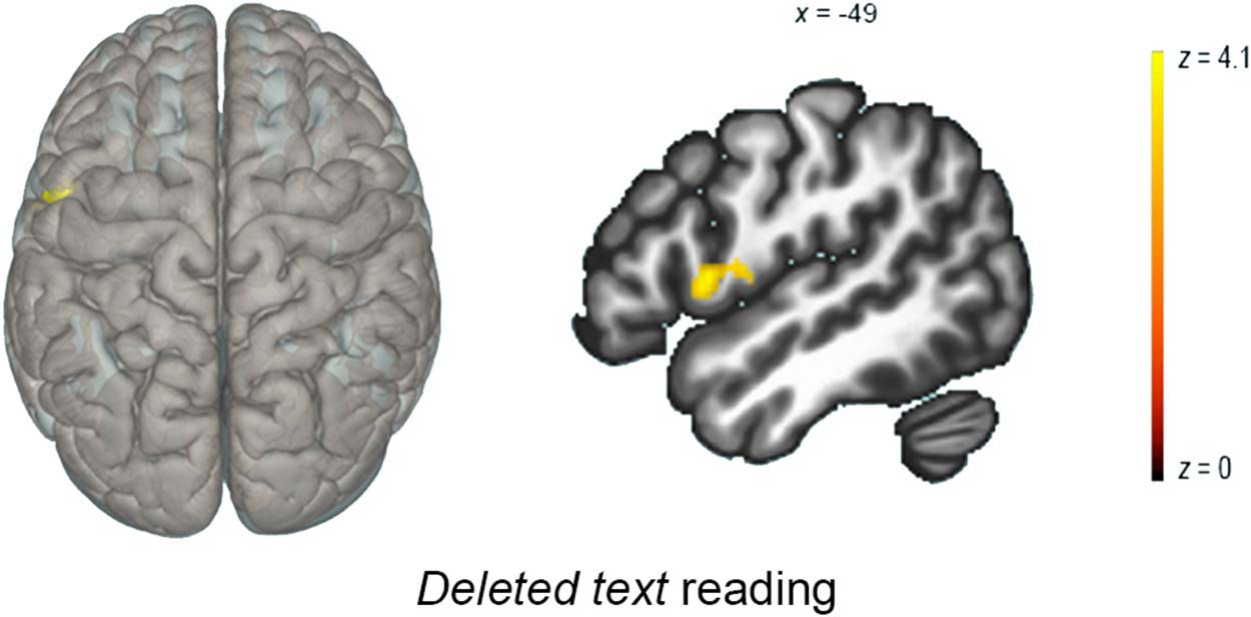
Seed to voxel analysis (DAN): typical readers. Seed-to-voxel analysis result. Cluster-level *p*-FDR corrected < .05. The seed is the DAN, contrast is EFs-based reading intervention > Math intervention, Deleted Post > Deleted Pre. Left: 3D brain render with a 50% transparency, superior view. Right: sagittal slice.

Deleted condition: Following intervention, TR who underwent the EFs-based reading training showed an increase in functional connectivity strength between the DAN and the left IFG while reading Deleted text, in comparison to TR who underwent the math intervention, as revealed using 2 × 2 (Time × Training) repeated measures ANOVA (T(41) = 3.54, cluster *p*-FDR < .05; Table 5, Figure 4). No differences were found between the connectivity patterns of the DAN in children with dyslexia when comparing the preintervention and the postintervention.

**Table 5.** Seed-to-voxel results

Seed	Group	Contrast	Functional connectivity	MNI coordinates			Cluster size (voxels)
				*x*	*y*	*z*	
DAN	TR	EFs-based int. > Math int., Deleted T2 > Deleted T1	Left inferior frontal gyrus	−48	10	8	313
	Children with dyslexia	–	–	–	–	–	–
VAN	TR	–	–	–	–	–	–
	Children with dyslexia	–	–	–	–	–	–
FP	TR	–	–	–	–	–	–
	Children with dyslexia	–	–	–	–	–	–
CO	TR	–	–	–	–	–	–
	Children with dyslexia	EFs-based int. Still T2 > Still T1	Right/Left precentral gyrus	−12	−24	72	178
		EFs-based int. > Math int. Still T2 > Still T1	Right lingual gyrus	20	−76	−4	299
			Right cuneus	10	−82	28	245

*Note*. Statistical threshold; cluster-level *p*-FDR corrected < .05, voxel-level uncorrected *p* < .001. Significant increases in functional connectivity were found in the CO and the DAN after the EFs-based reading intervention, but not the math intervention (active control group).

#### Ventral attention network.

No results were found in the VAN when comparing the connectivity pattern of this network with the rest of the brain before and after the trainings neither in Still nor Deleted conditions.

### EFs Networks

#### Cingulo-opercular network.

**Figure 5.**
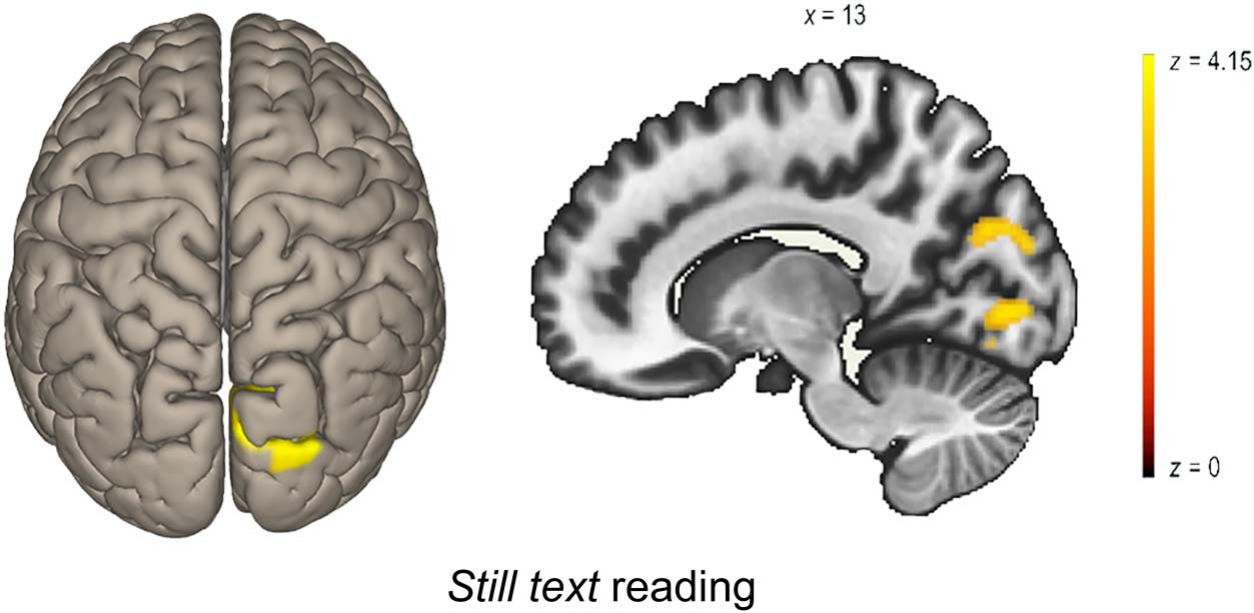
Seed-to-voxel analysis (CO): children with dyslexia. Seed-to-voxel analysis result. Cluster-level *p*-FDR corrected < .05. The seed is the CO network, contrast is EFs-based reading intervention > Math intervention, Still Post > Still Pre. Left: 3D brain render, superior view. Right: sagittal slice.

Still: Children with dyslexia who underwent the EFs-based reading intervention showed a higher functional connectivity between the cingulo-opercular (CO) network, the right cuneus, and the right lingual gyrus in the Still condition, in comparison to those undergoing math intervention (T(34) = 3.6, cluster *p*-FDR corrected < .05; see Table 5, Figure 5). In addition, in children with dyslexia who underwent the EFs-based reading intervention there was a higher functional connectivity between the CO and the right and left precentral gyrus after the intervention.

Deleted: No significant results were found in the seed-to-voxel analysis of the Deleted condition.

#### Fronto-parietal network.

No significant results were found when analyzing the FP network, neither in the Still nor the Deleted conditions.

### Random Classification

After randomly dividing the participants into four groups, the connectivity between the DAN and the rest of the brain was calculated. There was an increase in functional connectivity strength between the DAN and an occipital cluster comprising the left lingual gyrus in one of the random groups when comparing the pretest and posttest Still text reading conditions.

When comparing the pretest and posttest data of the random groups, no differences between random groups 1 and 2 were found (all groups included both TR and children with dyslexia undergoing both interventions). In the same vein, no significant differences were found between groups 3 and 4. Crucially, no differences between the randomly created groups were observed.

### Correlation Between Behavioral and Neuroimaging Data

**Figure 6.**
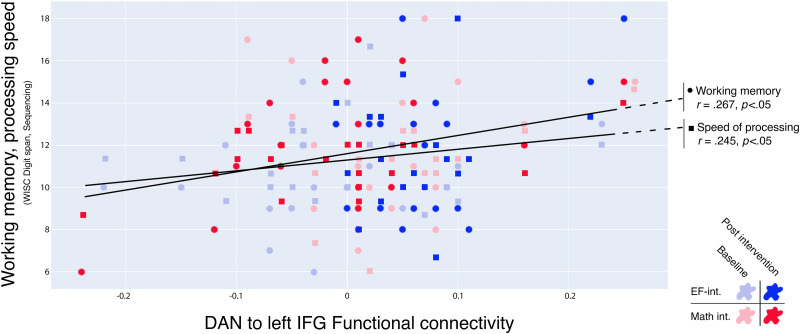
Correlation between the neuroimaging and behavioral results in Typical Readers. Higher synchronization between the DAN and the left IFG was related to increased performance in working memory and speed of processing.

**Figure 7.**
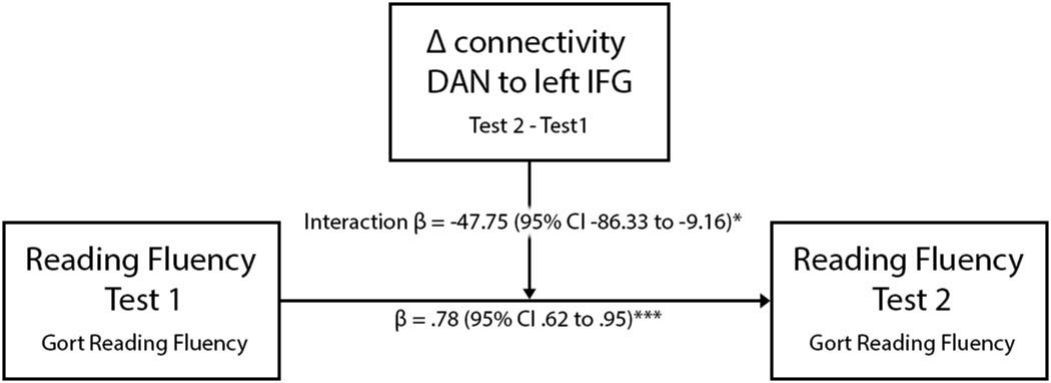
Simple moderation model. The change in connectivity between the DAN and the left IFG between the pretest and the posttest was a significant moderator of the reading improvement in Typical Readers, regardless of the intervention they received. Beta expressed as unstandardized regression coefficients. *p < .05, ***p < .001.

A positive correlation between the DAN and the left IFG during Deleted text reading and two cognitive measures was found in the group of TR—in the two training groups and the two time points: working memory (*r* = .27, *R*^2^ = .03, *p* < .05) and speed of processing (*r* = .24, *R*^2^ = .06, *p* < .05) (see Figure 6). This functional connectivity index was not correlated with working memory nor speed of processing in the group of children with dyslexia (working memory *r* = .15, *R*^2^ = .02, *p* = .24, speed of processing *r* = .04, *R*^2^ = .001, *p* = .71). In the same vein, the increase in connectivity strength DAN-left IFG after the intervention was found to be a significant moderator of the change in reading fluency for all TR (beta = 2.02, se = .94, *t*(3) = 2.14, *p* < .05; see Figure 7). In this moderation model, the main effect of both independent variables on the outcome variable were significant (GORT fluency before intervention *t* = 9.55, *p* < .001; delta DAN − left IFG *t* = 2.51, *p* < .05). This model was not significant in the group of participants with dyslexia.

**Figure 8.**
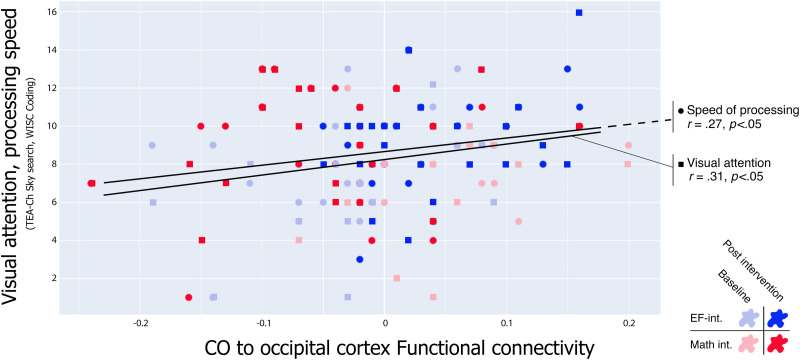
Correlation between the neuroimaging and behavioral results in children with dyslexia. Higher functional connectivity between the CO, the right lingual gyrus, and the right cuneus was related to increased performance in visual attention and processing speed tasks.

When exploring the behavioral correlates of the increased functional connectivity between the CO, the right cuneus, and the right lingual gyrus during the Deleted condition in children with dyslexia, a positive correlation was found with two cognitive variables: speed of processing (*r* = .27, *R*^2^ = .073, *p* < 0.05) and visual attention (*r* = .31, *R*^2^ = .095, *p* < 0.05; Figure 8). The increase in functional connectivity between the CO and the mentioned occipital regions was not a statistical predictor of the change in reading fluency in children with dyslexia (beta = .156, se = .13, *t*(3) = 1.3, *p* = .21). No significant correlations were found between the CO-right occipital cortex functional connectivity and behavioral measures in the TR group.

## DISCUSSION

Here, the specific effect of an EFs-based reading intervention versus a control math training were examined on the behavioral level and the neural correlates associated with EFs, visual attention, and reading in 8- to 12-year-old children with dyslexia and TR. In line with our hypotheses, we found significant improvements after the EFs-based reading intervention in reading rate and visual attention. However, we could not find a specific effect of the EFs-based reading intervention on relevant EFs (working memory, switching, inhibition), when comparing it to math training. On the neurobiological level, we found two different mechanisms related to the EFs-based reading intervention; in TR, higher connectivity was observed between the DAN and the left IFG. Children with dyslexia displayed an increase in integration between a cognitive control network (CO) and visual processing related areas. In both cases, the increase in connectivity positively correlated with performance in different cognitive tasks.

### Improvements in Reading and Visual Attention After the EFs-Based Reading Intervention

At the baseline, children with dyslexia displayed deficits in visual attention, EFs, phonological processing, word reading, and contextual reading when compared to TR. These impairments were not explained by reduced scores in standardized intelligence tests.

After the EFs-based reading intervention (compared to math intervention), improvements in children with dyslexia were apparent in both contextual reading rate and visual attention. Higher scores were observed as well in the TR group after the EFs-based reading intervention, but only in contextual reading rate (when compared to those undergoing math training). These results suggest that the EFs-based reading intervention effectively improves the reading skills and visual attention abilities of children with dyslexia. However, it was not possible to find a specific effect of the EFs-based reading intervention on EFs when comparing it to math intervention. Mathematical training, and arguably any kind of complex cognitive training, may involve one or more EFs given that these skills are the very basic psychological abilities underlying complex behavioral tasks—those that result from the combination of more basic mental processes (American Psychiatric Association, 2007). Mathematical reasoning, long-term planning, writing, or reading are some examples of these complex behavioral tasks that rely on the harmonic functioning of fundamental cognitive processes, many of which fall within the umbrella term Executive Functions.

The improvement in visual attention abilities after the EFs-based reading intervention in children with dyslexia confirms two of our assumptions. First, the EFs-based reading intervention triggers not only EFs but also basic features of visual processing (Horowitz-Kraus, Cicchino, et al., 2014). Second, the training of visual attention abilities may be of high relevance in children with dyslexia and could lead to improvements in reading (Vidyasagar, 2019).

This study presents evidence of improvements in visual attention and reading rate following an EFs-based reading intervention. Visual attention and EFs were found to be correlated with the intervention-related change in functional connectivity (in children with dyslexia). However, no causal relations were found between improvements in visual attention or EFs and improvements in reading ability. As aforementioned, the math intervention may have also targeted basic cognitive abilities within the EFs and visual attention domains as well as the EFs-based reading intervention, which could be the reason that prevented determining more specific effects of the EFs-based intervention. Another limitation was the use of a single visual attention measure (visual search or selective visual attention). The utilization of different visual attention tasks with a closer relation to decoding may result in the determination of better associations between improvement in visual attention and reading performance. Future intervention studies, including a waiting list (passive) control group might be able to draw robust links between the training of EFs and visual attention and improvements in reading. Alternatively, the investigation of nonlinguistic cognitive training aimed at improving EFs and/or visual attention can shed light on this issue.

Participants with dyslexia showed a significant improvement in reading accuracy after the EFs-based reading intervention (compared to the control training), but not in reading fluency or reading comprehension. This might be due to the control training utilized here (math) and the well-established importance of EFs in math performance (Bull & Lee, 2014; Cragg & Gilmore, 2014). It is possible that Math training improved EFs, which, per the current study’s hypothesis, had an effect on reading abilities. Future studies, including active and passive control groups may be able to test the effect of EFs-based reading training and visual training for dyslexia. Experimental studies investigating interventions for dyslexia with variable loads of EFs and visual attention (perhaps adopting a parametric approach with different subjects receiving different weights for each component) might enhance our understanding of the importance of each one of these factors in the treatment of dyslexia.

### Longer Response Times in the Fluency Task After the Intervention

Significant increases in response time were observed in the Still condition in all participants who underwent EFs-based reading intervention. Furthermore, only TR (not children with dyslexia) displayed longer response times in the posttest compared to the pretest after the math intervention. It has been previously suggested that in tasks involving EFs, longer response times are typically associated with superior performance (Goldhammer et al., 2014; Partchev, De Boeck, & Steyer, 2013; Su & Davison, 2019). On the contrary, longer response times in tasks that involve lower level processing indicate lower ability (Partchev et al., 2013). These studies imply that increased response times during reading tasks can reflect a deliberate effort by participants to perform adequately (greater involvement of EFs). The present results confirmed this hypothesis, given the significant association between longer response times (when comparing the pretest and the posttest) and enhanced performance in the posttest. Here, a significant correlation was found between pre-post changes in inhibition and pre-post changes in response time to the Still condition in all participants. That is, the participants showing larger score improvements in inhibition after the intervention displayed longer response times after the intervention, which is in line with the presented explanation. In this vein, longer response times after the intervention may be related to increases in cognitive inhibition, which is involved in response monitoring (Kilian, Bröckel, Overmeyer, Dieterich, & Endrass, 2020). In the present study, all groups who were expected to demonstrate reading improvement in response to the EFs-based reading intervention, including both typically developing readers and those with dyslexia, exhibited longer response times. In contrast, the group of children with dyslexia who received the Math intervention did not improve, which is consistent with the presented post hoc explanation.

Notably, this effect was not observed in the Deleted condition, where no significant differences in response time nor accuracy were found for any group. It is plausible that participants dedicated more time to ensure accuracy in the Still condition due to a reduced sense of urgency to respond rapidly, while the accelerated presentation pace of the Deleted condition induced an equally fast response.

### Greater Synchronization Between EFs Networks and Visual Processing Regions After the EFs-Based Reading Intervention

Stronger functional connectivity was found in children with dyslexia between the CO, the right lingual gyrus, and the right cuneus after the EFs-based reading training. The cuneus and lingual gyrus comprise the medial occipital lobe and have a role in basic and higher level visual processing (Allison et al., 1993; Mai & Paxinos, 2012; Palejwala et al., 2021). More specifically, the cuneus and lingual gyrus seem to have special relevance in visual memory, linguistic processing (written words), direction discrimination, and motion perception (Palejwala et al., 2021). Taking into account the role of these areas in word decoding and movement-related features of visual processing, it seems reasonable to assume that the fast-paced deleted letters characterizing the EFs-based reading intervention triggered a higher synchronization between the mentioned visual processing regions and a higher order cognitive control network in order to achieve better performance at the task. This increase in functional connectivity is interpreted as an indicator of higher integration of the basic (and not-so-basic) cognitive processes underlying fluent reading, that is, visual attention, working memory, speed of processing, and more (Dehaene, 2009; Sporns, 2012). Here, a positive correlation was found between these regions’ connectivity and the scores in visual attention and speed of processing.

Despite the major role of the left fusiform gyrus (or VWFA) in word decoding, we did not find an association between this region and the EFs-based intervention-related cognitive gain. Here, children with dyslexia displayed an increase in connectivity strength between the CO and occipital areas involved in visual processing, but not the VWFA. The lingual gyrus and the right cuneus possess certain processing features that allow them to engage in the primary processing of visual information, further feeding into the (hierarchichally superior) occipito-temporal VWFA. The present results suggest that the decoding deficit in dyslexia may arise in early stages of visual processing, which is not in synchrony with higher order cognitive control networks. This beneficial pattern of connectivity was reinforced after the EFs-based reading training. How are the interactions between the VWFA and the rest of the brain affected in dyslexia and how can a treatment address this connections is a matter of further research.

Recent studies suggest that one of the mechanisms of brain maturation along development is a higher synchronization between whole-brain networks and versatile neural hubs such as the IFG (Hermosillo, 2022; Wierenga et al., 2016). The medial occipital lobe, and specifically the bilateral lingual gyri, seem to adopt a major role as an association hub along development (Z. Chen, Liu, Gross, & Beaulieu, 2013; Oldham & Fornito, 2019). By boosting the synchrony between the CO and the medial occipital lobe, the EFs-based reading intervention might be enforcing a pattern of brain maturation in children with dyslexia (Hermosillo, 2022), which might help them overcome the asynchronization reported earlier (Breznitz & Misra, 2003) and reduce neural noise in their sensory systems (Hancock et al., 2017).

### Greater Engagement of Attention and Linguistic/Multimodal Regions in TR After the EFs-Based Reading Intervention

A different neuroplasticity mechanism was found in TR children who underwent the EFs-based reading intervention. In this subgroup, higher functional connectivity was detected between the DAN and the left IFG while reading deleted text. The left IFG is associated with phonological and semantic functions (Klaus & Hartwigsen, 2019). The role of this region in word comprehension and production is undisputed (Costafreda et al., 2006; Klaus & Hartwigsen, 2019). Furthermore, it is now well established that the left IFG is a neuronal hub playing a role in multiple interrelated lower and higher order cognitive operations such as multisensory integration (Li, Seger, Chen, & Mo, 2020; Pulvermüller, 2013), verbal working memory (Costafreda et al., 2006; Emch, von Bastian, & Koch, 2019), creativity (Khalil, Karim, Kondinska, & Godde, 2020), and inhibitory control (Swick, Ashley, & Turken, 2008; Tomiyama et al., 2022). In the same vein, the bilateral inferior frontal gyri participate in distinct large-scale networks such as the language network (Gao, Guo, Liu, Mo, & Wang, 2020; Pulvermüller, 2013; Tomasello, Garagnani, Wennekers, & Pulvermüller, 2017), the VAN (Bernard et al., 2020; Corbetta & Shulman, 2002), and the limbic network (Cha et al., 2016; Rolls et al., 2020), with broad connections to the temporal and parietal cortices (Nakae et al., 2020). The increase in strength of functional connectivity between the DAN and the left IFG found after the EFs-based reading intervention suggests that our training paradigm enforced in TR a maturation in brain network interactions typical of the late childhood period (Oldham & Fornito, 2019; Sporns, 2010).

Higher synchrony between the DAN and the left IFG was related to higher scores in verbal working memory, which is a cognitive ability that heavily relies on the frontal lobe and the language network, including the left IFG (Dehaene, 2009; Emch et al., 2019). The involvement of the DAN in visuo-attentional processing (Szczepanski & Kastner, 2013; Zhao, Wang, Huang, & Liang, 2022) makes it a major candidate to contribute to the reading process, and several studies have indeed highlighted the role of (especially posterior) DAN areas in reading (Cohen, Dehaene, Vinckier, Jobert, & Montavont, 2008; Qian, Bi, Wang, Zhang, & Bi, 2016). However, the specific role of the different areas comprising the dorsal attention system in the processing of printed words is not yet completely clear. Our results suggest that higher synchrony between the DAN and the left IFG is related to higher cognitive performance in speed of processing, working memory and reading in TR, and that the EFs-based reading intervention is capable of inducing increases in functional connectivity between these areas.

### No Significant Differences When Randomly Dividing the Sample

Here, we included an additional analysis in which random groups were created and the seed-to-voxel analysis was repeated in order to check for the validity of our results. One pre-post test contrast reached significance in one of the subgroups. An increase in connectivity after the training in both TR and participants with dyslexia undergoing either intervention could be representing a mechanism common to both interventions. Alternatively, it could be a neurobiological change related to normative development. In any case, it remains unanswered why this change in connectivity is visible in one of the subgroups only, and this result is arguably an indicator of the spurious effects that can be found performing fMRI analyses when running undirected contrasts. Crucially, no differences were found between any of the random groups, arguing in favor of the empirical strength of the results presented in the present study.

### Limitations

Recent benchmarking papers have criticized the poor reliability of BWAS (Baykara, Könen, Unger, & Karbach, 2021; Marek et al., 2022). Here, we did not explicitly conduct a BWAS, but we did include univariate prediction models for brain-behavioral phenotypes. Even though we tried addressing this limitation by performing an extra control analysis (group permutation), the need for larger sample sizes in order to increase the reliability and generalizability of the associations in the field of cognitive neuroscience is an unavoidable reality. Furthermore, current recommendations highlight the need to move toward individualized treatment approaches when addressing cognitive traits in clinical populations (Baykara et al., 2021). The utilization of different active as well as passive (waiting list) control groups might produce better outcomes when studying the neuropsychological effect of an intervention program; we were not able here to isolate the effect of the EFs-reading intervention on executive functions themselves because of the recruitment of EFs in mathematical thinking. In summary, individualized programs combining features of the EFs-based reading intervention (and other phonological and visual trainings) might be the most fruitful research direction for the development of treatment plans for dyslexia in upcoming years.

### Conclusion

The EFs-based reading intervention improved the reading skills of children with dyslexia and TR, and two different neuroplasticity mechanisms were observed. In TR, the development of more mature brain connectivity patterns between a large-scale network (i.e., DAN) and a multimodal hub (i.e., left IFG) seemed to be mediating the reading fluency improvement. In children with dyslexia, the synchronized activity of visual processing areas (which have also been suggested to act as integration hubs) (Z. Chen et al., 2013) and performance monitoring cognitive control systems was related to higher performance in low-level (i.e., visual attention) and higher order cognitive tasks (i.e., working memory) that are foundational for reading. These findings point at the importance of addressing EFs and visual attention in the development of interventions aimed at the improvement of reading in dyslexia.

## ACKNOWLEDGMENTS

This study was supported by the National Institute of Child Health and Human Development (R01 HD086011; PI: Horowitz-Kraus).

## AUTHOR CONTRIBUTIONS

Nikolay Taran: Formal analysis; Methodology; Writing – original draft; Writing – review & editing. Rola Farah: Formal analysis; Writing – original draft. Carmel Gashri: Formal analysis; Writing – original draft. Ester Gitman: Formal analysis. Keri Rosch: Funding acquisition; Writing – original draft; Writing – review & editing. Bradley L. Schlaggar: Funding acquisition; Writing – original draft; Writing – review & editing. Tzipi Horowitz-Kraus: Conceptualization; Data curation; Formal analysis; Funding acquisition; Investigation; Methodology; Project administration; Resources; Supervision; Validation; Visualization; Writing – original draft; Writing – review & editing.

## FUNDING INFORMATION

Tzipi Horowitz-Kraus, National Institute of Child Health and Human Development (https://dx.doi.org/10.13039/100000071), Award ID: R01HD086011-05.

## TECHNICAL TERMS

Executive functions (EFs): An umbrella term for a set of top-down cognitive abilities involved in reasoning, problem-solving, and planning.
Visual word form area (VWFA): Refers to a specific neural region situated in the left fusiform gyrus (inferior occipito-temporal area) whose processing features facilitate the recognition and decoding of written words.
Cingulo-opercular network (CO): Includes the dorsal anterior cingulate cortex, the anterior insula, and the dorsolateral prefrontal cortex. It underlies the control of goal-directed behavior through sustained cognitive control.
Fronto-parietal network (FP): Involved in the initiation of task sets and the coordination of whole-brain network activity, playing a key role in complex cognitive abilities such as reading or long-term memory.
Ventral attention network (VAN): Comprises the ventral frontal cortex and temporoparietal junction and is involved in bottom-up (stimulus-driven) attention processes.
Dorsal attention network (DAN): Comprises the inferior frontal gyrus and the frontal eye fields and is thought to the neurobiological support of endogenous attention.
Event-related potentials (ERPs): The name given to small voltages generated in specific brain structures in response to certain stimuli.
Visual attention: Defined as the ability to select the appropriate stimulus from a cluttered visual scene. Importantly, visual attention comprises multiple interacting top-down and bottom-up processes.
